# Is the Health of Older Americans With a GED Equivalent to Their Peers With a High School Diploma?

**DOI:** 10.1177/00914150231208685

**Published:** 2023-10-24

**Authors:** Esme Fuller-Thomson, Robin Grossman, Andie MacNeil

**Affiliations:** 1 Factor-Inwentash Faculty of Social Work, 7938 University of Toronto, Toronto, Ontario, Canada; 2 Institute for Life Course and Aging, 7938 University of Toronto, Toronto, Ontario, Canada; 3 Department of Family and Community Medicine & Faculty of Nursing, 7938 University of Toronto, Toronto, Ontario, Canada

**Keywords:** activities of daily living (ADLs), cognition, disability, health, ‌General Educational Development (GED)

## Abstract

The aim of this study was to identify differences in the prevalence and odds of cognitive impairment, hearing impairment, vision impairment, limitations in activities of daily living (ADLs), and ambulation limitations among three groups of older American adults: high school dropouts, General Educational Development (GED) recipients, and high school graduates. This study used secondary analysis of the nationally representative 2017 American Community Survey. The sample included 20,489 GED recipients, 154,892 high school graduates, and 49,912 high school dropouts. Our findings indicate that there is a gradient in health outcomes among older Americans, with the highest prevalence and odds of cognitive impairment, hearing impairment, vision impairment, ADL limitations, and ambulation limitations among high school dropouts, followed by GED recipients, and the lowest among high school graduates. Although GED recipients have better health outcomes than high school dropouts, there is still a significant disparity in health status between GED recipients and high school graduates.

The GED is a series of standardized tests that are used to determine if individuals have knowledge equivalent to that of high school graduates (Heckman et al., 2011). The GED was originally developed in 1942 to address the needs of military service personnel who had enlisted in the Second World War before completing high school (Heckman et al., 2011). The GED has continued to evolve over the past 80 years. Most recent available data indicate that approximately 20 million people have passed the GED test since its inception (American Council on Education, 2014).

Higher levels of educational attainment are associated with better health outcomes, such as lower rates of morbidity and mortality (Grossman, 2015; Kaestner et al., 2020). While considerable research has emphasized differences in health status between those with and without a university degree (Case & Deaton, 2015; Case & Deaton, 2017), as well as high school graduates and high school dropouts (De Ridder et al., 2013), less research has examined differential health outcomes among those with a GED (Caputo, 2005; Zajacova & Everett, 2014). There is an underlying assumption that the GED is equivalent to a high school diploma (Zajacova, 2012). Indeed, population statistics on educational attainment usually combine high school graduates with GED recipients (National Center for Education Statistics [NCES], 2021), thereby obscuring any potential health differences between these groups.

Reviewing the available literature on the health of GED recipients indicates that they typically have worse health outcomes when compared to high school graduates, suggesting that these populations may not be equivalent as previously assumed. When compared to high school graduates, GED recipients have higher rates of obesity (Zajacova & Everett, 2014), are more likely to experience depression (Caputo, 2005), and are more likely to engage in unhealthy behaviors, such as cigarette smoking (Kurti et al., 2016), heavy alcohol use (Zajacova, 2012), and excessive cannabis use (Gonzalez et al., 2016). Zajacova (2012) compared the health status of high school dropouts, high school graduates, and GED recipients among adults aged 30–65 based on 25 health indicators, including chronic health conditions (e.g., hypertension, asthma, and diabetes), acute illness (e.g., common cold), and general health indicators (e.g., functional status), and found that GED recipients had a higher prevalence of all 25 health indicators when compared to high school graduates. GED recipients are also less likely to be up-to-date with recommended preventive medical procedures, such as mammograms, pap smears, dental cleanings, and flu shots than their peers who graduated from high school (Song & Hsu, 2008). These health disparities have implications throughout the lifetime, as evidenced by higher rates of self-reported disability, incident IADL limitations, and premature mortality among GED recipients compared to high school graduates (Liu et al., 2013; Rogers et al., 2010).

While the available research provides an important foundation to understand differential health outcomes among GED recipients, further research is warranted to understand how these health disparities may unfold across the life course, with particular consideration for health outcomes in older adulthood. The life course perspective provides a theoretical lens through which health outcomes can be understood as the accumulation of an individual's experiences, including the impact of various sociodemographic factors, all of which are nested within a broader structural and historical context (Halfon & Hochstein, 2002). In particular, the life course perspective has been used to understand how socioeconomic conditions in early life, such as educational attainment, influence health outcomes in older adulthood (Wagner et al., 2022). Furthermore, it provides a helpful framework for understanding how health disparities are impacted by cohort differences. This is of particular importance when examining the effects of education on health outcomes among older adults, as educational attainment among this population was largely impacted by cohort differences, such as the impact of the Great Depression and World War II on life trajectories (Elder & George, 2016). Utilizing a life course perspective, the objective of the current study is to identify differences in the prevalence and odds of cognitive impairment, hearing impairment, vision impairment, limitations in ADLs, and ambulation limitations among three groups of American older adults: high school dropouts, GED recipients, and high school graduates, stratified into three age cohorts (65–74, 75–84, and 85+). We hypothesize that (1) GED recipients will have a significantly higher prevalence and odds of cognitive impairment, hearing impairment, vision impairment, ADL limitations, and ambulation limitations than high school graduates; and (2) GED recipients will have a significantly lower prevalence and odds of cognitive impairment, hearing impairment, vision impairment, ADL limitations, and ambulation limitations than high school dropouts.

## Methods

### Sample

As has been discussed elsewhere (Deng et al., 2021), this study used data from the 2017 American Community Survey (ACS-2017). The ACS is an annual survey that replaces the long form of the decennial census. The ACS uses a nationally representative sample of Americans living in communities and in group quarters (e.g., long-term care facilities). Response rates were 94% in 2017 (United States Census Bureau, 2019). The sample for the current analyses was restricted to 225,293 older Americans (age 65 and older)**,** of whom 20,489 had their GED, and 154,892 had a regular high school diploma (neither group had any post-high school education), and 49,912 who had finished grade 8 but not completed high school (hereafter referred to as “high school dropouts”). Institutional review board's approval was not needed as only publicly available nonidentifiable data were used.

### Measures

*Cognitive Impairment* was assessed based on the following question: “Because of a physical, mental, or emotional condition, does this person have serious difficulty concentrating, remembering, or making decisions?” (Yes/no).

*Hearing Impairment* was assessed based on the question “Is this person deaf or does he/she have serious difficulty hearing?” (Yes/no).

*Vision Impairment* was assessed based on the following question: “Is this person blind or does he/she have serious difficulty seeing even when wearing glasses?” (Yes/no).

*Limitations in Activities of Daily Living (ADLs)* were assessed based on the following question: “Does this person have difficulty dressing or bathing?” (Yes/no).

*Ambulation Limitations* were assessed based on the following question: “Does this person have serious difficulty walking or climbing stairs?” (Yes/no).

These questions were identical for those living in the community and institutionalized settings, with the exception of the change from the first person in the former to the third person in the latter (i.e., “do you…” becomes “does this person…”). Surveys were completed independently by capable respondents or by caregivers or nursing home staff members (Torrieri, 2014).

*Key Exposure of Interest:* Highest level of education completed. This measure was based on the question “What is the highest degree or level of school this person has completed?” Three levels of education were included in this study: GED recipients (reference category), high school graduates, and high school dropouts (grade 9, 10, 11, 12 [no diploma]).

*Control variables*: Sex was based on self-report (male, female). Age was divided into 10-year periods for the age groups (65–74), (75–84), and (≥85) to generate prevalence estimates for bivariate analyses. For logistic regression analyses, age was entered by each year categorically (top coded at 96). This was performed to address the non-linear relationship between age and disabilities. Race/ethnicity was divided into 6 categories: non-Hispanic White, non-Hispanic Black, non-Hispanic American Indian/Alaskan Native, non-Hispanic Asian, non-Hispanic Native Hawaiian/Pacific Islander, and Hispanic of any race.

The ACS measures an individual's household poverty status by comparing a household's total income against a pre-determined income poverty threshold for households of that size and composition. The income variable was categorized into the following: <poverty line, 100%-199%, 200%-299%, 300%-399%, 400%-499%, 500% and more of the poverty line, and a missing data category.

### Statistical Analysis

Prevalence data were generated for each age cohort (i.e., 65–74, 75–84, 85+, and for all respondents aged 65+). Logistic regression analyses were conducted for the entire sample aged 65 and older with the outcome of cognitive impairment and the key variable of interest being the level of education. In the logistic regression analyses, race/ethnicity, age (by year), sex, and poverty level were entered as categorical variables. This process was repeated for each of the other outcomes of interest (hearing impairment, vision impairment, ADL limitations, and ambulation limitations), resulting in a total of five different logistic regression analyses for the combined sample of those aged 65 and older. Similar logistic regression analyses were conducted for each of the three other age categories (i.e., 65–74; 75–84; 85+) and for men aged 65 + and women aged 65 + . All data were weighted to adjust for non-response and differential selection probabilities (United States Census Bureau, 2010). All sample sizes are represented in their unweighted form. All analyses were conducted using IBM SPSS 25.

## Results

Table 1 presents the prevalence of each of the five types of impairment by the highest level of education completed (high school dropouts, GED recipients, and high school graduates) for four age cohorts (i.e., 65–74, 75–84, 85+, and for all respondents aged 65+). Those with a GED had a lower prevalence of each of the five forms of impairment than high school dropouts for all four age cohorts, with one exception (i.e., hearing impairment, aged 75–84).

**Table 1.** Prevalence of Cognitive Impairment, Hearing Impairment, Vision Impairment, Limitations in Activities of Daily Living, and Ambulation Limitations by Education Level (High School Dropout/GED recipient/High School Graduate) by Age Cohort.

Variables	Unweighted sample	% With cognitive impairment	% With hearing impairment	% With vision impairment	% With limitations in activities of daily living	% With ambulation limitations
Aged 65–74						
High School Dropout	21,343	13.0	14.2	8.8	10.2	30.1
GED Recipient	12,268	9.8	13.9	6.7	6.9	24.8
High School Graduate	80,374	6.4	9.7	4.4	5.7	17.9
Total Sample Size	113,985	8.0	11.0	5.5	6.7	21.0
Linear by linear association		1069.38	435.95	662.90	545.85	1659.55
P-value		<.001	<.001	<.001	<.001	<.001
**Aged 75**–84						
High School Dropout	17,980	16.6	22.7	11.1	15.3	37.5
GED Recipient	6,363	11.9	23.4	8.6	11.1	31.5
High School Graduate	49,982	11.8	17.6	6.9	11.2	28.1
Total Sample Size	74,325	13.0	19.3	8.1	12.2	30.7
Linear by linear association		259.01	262.46	318.71	197.18	550.48
P-value		<.001	<.001	<.001	<.001	<.001
**Aged 85+**						
High School Dropout	10,589	31.7	40.9	20.5	33.0	57.8
GED Recipient	1,858	23.6	37.4	13.6	21.5	47.5
High School Graduate	24,536	28.5	33.8	15.8	30.7	53.6
Total Sample Size	36,983	29.1	36.0	17.0	30.9	54.5
Linear by linear association		29.75	159.27	104.12	9.94	42.74
P-value		<.001	<.001	<.001	.002	<.001
**Aged 65+**						
High School Dropout	49,912	18.1	22.7	12.0	16.6	38.4
GED Recipient	20,489	11.8	19.1	7.9	9.6	29.1
High School Graduate	154,892	11.6	16.0	7.0	11.4	26.9
Total Sample Size	225,293	13.1	17.8	8.2	12.4	29.6
Linear by linear association		1282.68	1174.10	1190.42	800.19	2326.86
P-value		<.001	<.001	<.001	<.001	<.001

Source: American Community Survey 2017. GED: General Educational Development.

When the younger cohorts (i.e., 65–74 and 75–84) and all those aged 65 + together were considered, those with a GED had a *higher* prevalence of each of the five forms of impairment than that of high school graduates with only two exceptions (i.e., limitations in ADLs for those 65 + and those aged 75–84). However, the pattern was dramatically different in the 85 + cohort: for every outcome except for hearing loss, those with a GED had substantially *lower p*revalence of impairment compared to high school graduates.

As shown in Table 2, the age-sex-race and poverty-adjusted odds of each of the five types of impairment was consistently higher for high school dropouts compared to those with a GED for all age cohorts and all outcomes (with one exception of hearing impairment among men aged 65+). However, in some cases, these elevated odds failed to reach statistical significance (e.g., ADL limitations for those aged 75–84 and female respondents aged 65+; hearing impairment for those aged 65–74 and 75–84; and all but ADL limitations among men aged 65+).

**Table 2.** Odds Ratio of Cognitive Impairment, Hearing Impairment, Vision Impairment, Limitations in Activities of Daily Living, and Limitations in Ambulation by Education Level (High School Dropout/GED Recipient/High School Graduate) Controlling for Sex, Race, Age in Years, and Poverty Level.

Variables	Unweighted sample	Odds ratio (95% CI) of cognitive impairment	Odds ratio (95% CI) of hearing impairment	Odds ratio (95% CI) of vision impairment	Odds ratio (95% CI) of limitations in activities of daily living	Odds ratio (95% CI) of ambulation limitations
Aged 65+ (both sexes)						
High school dropout	49,428	1.11 (1.06, 1.17)	1.06 (1.02, 1.11)	1.17 (1.10, 1.25)	1.15 (1.09, 1.22)	1.08 (1.04, 1.12)
Ged recipient	20,287	1.00 (Ref)	1.00 (Ref)	1.00 (Ref)	1.00 (Ref)	1.00 (Ref)
High school graduate	153,932	0.79 (0.76, 0.83)	0.76 (0.73, 0.80)	0.78 (0.73, 0.82)	0.90 (0.86, 0.95)	0.75 (0.72, 0.77)
Total sample size	223,647					
Model chi-square (df)		20,910 (45)	19,054 (45)	7179 (45)	32,286 (45)	28,934 (45)
*p*-value		<.001	<.001	<.001	<.001	<.001
Pseudo *R*^2^		0.166	0.134	0.073	0.255	0.173
Aged 65+ (male only)						
High school dropout	21,253	1.08 (1.00, 1.17)	0.99 (0.94, 1.05)	1.09 (1.00, 1.19)	1.26 (1.15, 1.38)	1.04 (0.98, 1.10)
GED recipient	10,100	1.00 (Ref)	1.00 (Ref)	1.00 (Ref)	1.00 (Ref)	1.00 (Ref)
High school graduate	58,603	0.78 (0.73, 0.84)	0.75 (0.71, 0.79)	0.82 (0.75, 0.89)	1.03 (0.95, 1.12)	0.76 (0.72, 0.80)
Total sample size	89,956					
Model chi-square (df)		6076 (44)	5231 (44)	2269 (44)	9112 (44)	8506 (44)
*p*-value		<.001	<.001	<.001	<.001	<.001
Pseudo *R*^2^		0.128	0.087	0.061	0.201	0.135
Aged 65+ (female only)						
High school dropout	28,175	1.13 (1.05, 1.21)	1.16 (1.08, 1.25)	1.22 (1.12, 1.32)	1.05 (0.98, 1.14)	1.11 (1.05, 1.17)
GED recipient	10,187	1.00 (Ref)	1.00 (Ref)	1.00 (Ref)	1.00 (Ref)	1.00 (Ref)
High school graduate	95,329	0.79 (0.74, 0.85)	0.80 (0.74, 0.85)	0.75 (0.69, 0.81)	0.81 (0.76, 0.87)	0.74 (0.70, 0.77)
Total sample size	133,691					
Model chi-square (df)		14,884 (44)	11,635 (44)	4972 (44)	22,863 (44)	19,562 (44)
*p*-value		<.001	<.001	<.001	<.001	<.001
Pseudo *R*^2^		0.189	0.148	0.082	0.282	0.188
Aged 65–74						
High school dropout	21,111	1.14 (1.06, 1.23)	1.05 (0.98, 1.12)	1.14 (1.04, 1.25)	1.25 (1.14, 1.36)	1.11 (1.05, 1.17)
GED recipient	12,134	1.00 (Ref)	1.00 (Ref)	1.00 (Ref)	1.00 (Ref)	1.00 (Ref)
High school graduate	79,806	0.65 (0.61, 0.70)	0.73 (0.68, 0.77)	0.68 (0.63, 0.74)	0.82 (0.76, 0.89)	0.67 (0.64, 0.71)
Total sample size	113,051					
Model chi-square (df)		4441 (23)	4362 (23)	1655 (23)	6357 (23)	6894 (23)
*p*-value		<.001	<.001	<.001	<.001	<.001
Pseudo R^2^		0.090	0.076	0.042	0.141	0.092
Aged 75–84						
High school dropout	17,803	1.19 (1.08, 1.30)	1.02 (0.95, 1.10)	1.16 (1.05, 1.29)	1.08 (0.98, 1.19)	1.08 (1.02, 1.16)
GED recipient	6,306	1.00 (Ref)	1.00 (Ref)	1.00 (Ref)	1.00 (Ref)	1.00 (Ref)
High school graduate	49,697	0.88 (0.81, 0.96)	0.76 (0.71, 0.81)	0.77 (0.70. 0.85)	0.85 (0.77, 0.92)	0.77 (0.73, 0.82)
Total sample size	73,806					
Model Chi-Square (df)		4433 (23)	2870 (23)	873 (23)	7186 (23)	5161 (23)
*p*-value		<.001	<.001	<.001	<.001	<.001
Pseudo *R*^2^		0.108	0.061	0.027	0.177	0.095
Aged 85+						
High school dropout	10,514	1.16 (1.03, 1.31)	1.20 (1.08, 1.33)	1.44 (1.24, 1.66)	1.27 (1.12, 1.44)	1.19 (1.07, 1.32)
GED recipient	1,847	1.00 (Ref)	1.00 (Ref)	1.00 (Ref)	1.00 (Ref)	1.00 (Ref)
High school graduate	24,429	1.04 (0.93, 1.17)	0.87 (0.79, 0.96)	1.08 (0.94, 1.24)	1.22 (1.08, 1.38)	1.02 (0.93, 1.13)
Total sample size	36,790					
Model chi-square (df)		3781 (25)	1449 (25)	876 (25)	6920 (25)	3762 (25)
*p*-value		<.001	<.001	<.001	<.001	<.001
Pseudo *R*^2^		0.141	0.054	0.040	0.244	0.131

*Source:* American Community Survey 2017. GED: General Educational Development.

High school graduates had statistically significant lower odds of all five impairments compared to GED recipients for every age and gender cohort examined except for those aged 85 and older and ADL limitations among those aged 65 and older. Among those aged 85 and older, high school graduates had significantly *lower* odds of hearing impairment, significantly *higher* odds of ADL limitations and the odds of cognitive impairment, vision impairment and ADL impairment were comparable.

## Discussion

### Summary of Key Findings

The results of this nationally representative study of almost a quarter of a million American older adults (n = 225,293) indicate a gradient in health outcomes by level of educational attainment. Among older adults aged 65 and older, the odds of cognitive impairment, hearing impairment, vision impairment, ADL limitations, and ambulation limitations were consistently lower among high school graduates and higher among high school dropouts when compared to GED recipients, with very few exceptions. This indicates that although older adult GED recipients generally have better health outcomes than high school dropouts, they should not be assumed to be equivalent to their high school graduate counterparts.

When the analysis was limited to female respondents only, the same gradient was observed for cognitive impairment, hearing impairment, vision impairment, and ambulation limitations. Upon examining ADL limitations among female respondents only, there was no statistically significant difference between high school dropouts and GED recipients, while high school graduates outperformed both groups. For cognitive impairment, hearing impairment, vision impairment, and ambulation limitations among males only, there were no statistically significant differences between high school dropouts and GED recipients, while high school graduates still had lower odds of each outcome. For ADL limitations among males only, there was no statistically significant difference between high school graduates and GED recipients, while high school dropouts still had higher odds.

There was also variation when the analysis was separated into age cohorts. Older adults aged 65–74 showed a consistent gradient for cognitive impairment, vision impairment, ADL limitations, and ambulation limitations, in which high school dropouts had higher odds of experiencing each health outcome when compared to GED recipients, while high school graduates had lower odds when compared to GED recipients. The only exception for this age group was hearing impairment, for which there was no statistically significant difference between high school dropouts and GED recipients. Among those aged 75–84, the same gradient was present for cognitive impairment, vision impairment, and ambulation limitations, but it was not present for hearing impairment and ADL limitations, where there was no statistically significant difference between high school dropouts and GED recipients.

The gradient identified in the entire sample was not present among those aged 85 and older. In this group, high school dropouts consistently had higher odds of experiencing each health outcome when compared to GED recipients; however, high school graduates only had significantly lower odds for hearing impairment, while they had significantly higher odds of ADL limitations, and did not differ on the other three forms of impairment. This suggests that within the oldest age cohort, there may not be prominent differences in health status between GED recipients and high school graduates that were present in younger age cohorts. The life course perspective may help explain this exception to the pattern. The oldest cohort in the current study (85+) was born in 1932 or earlier and therefore grew up during the Great Depression and may have served in the Second World War. As a result, many respondents in this cohort likely experienced more pressure to leave school prior to receiving a high school diploma in order to serve in the military. The GED was developed specifically to support veterans who gained critical life experiences from serving in the military but lacked the credentials of a high school diploma due to leaving school early (Quinn, 2014). However, by the 1960s, the GED began to shift away from being an alternative for veterans and was instead purported by the federal government as a way for any adolescent or young adult to get high school credentials, regardless of life experience or reasons for not completing high school. Indeed, between 1953 and 1966, the percentage of GED test-takers who were veterans declined from 60% to 20% (Humphries, 2014). As the GED program expanded to be more inclusive to high school dropouts, there were many individuals with literacy levels below the sixth-grade level who were still able to train for and successfully pass the test, ultimately receiving high school equivalency despite a lack of equivalent experience (Quinn, 2014). This may explain some of the observed differences in the current study, as the youngest cohort in the current study represents a very different subset of GED recipients than the oldest cohort.

### Cognitive Impairment

The findings of this study support existing research that has demonstrated high school completion reduces the likelihood of cognitive impairment compared to individuals who did not complete high school (Marden et al., 2017). This study also expands upon existing research on education and cognitive impairment by separating GED recipients from high school graduates. The current study found that high school graduates were less likely to have cognitive impairment than those with a GED, suggesting that these populations may not be as equivalent as they are often presumed.

There are several factors that may make GED recipients more vulnerable to cognitive impairment than high school graduates. As per the cognitive reserve hypothesis, educational attainment throughout the lifespan is thought to be one of the mechanisms that increase cognitive reserve, ultimately allowing the brain to better tolerate age-related changes and making these individuals less likely to develop dementia in early older adulthood (Meng & D’Arcy, 2012). In other words, the mental stimulation of early education builds cognitive reserve, better maintaining cognitive function in later life. This may partially explain why high school dropouts do worse in cognitive health outcomes, as they may have lacked the same mental stimulation as their counterparts who completed school, resulting in a lower cognitive reserve. It is possible that the same phenomenon is occurring for GED recipients, and the supposed equivalency between GED recipients and high school graduates fails to consider the differences in their experiences of years in formal education.

GED recipients may also have higher odds of dementia than high school graduates because of differences in their midlife experiences. Although GED recipients and high school graduates are generally equivalent in their cognitive skill abilities, it is their differences in non-cognitive skills, such as discipline and self-efficacy, that may create challenges for GED recipients when it comes to performing the same as their high school graduate counterparts, often limiting their ability to thrive in their work environments (Heckman & Rubinstein, 2001). Multiple studies have indicated that GED recipients perform worse in the labour market than those with a high school diploma (Heckman & LaFontaine, 2006; Tyler, 2003; Tyler et al., 2003). Poorer performance in the labour market may lead to greater stressors in midlife, such as employment stress and financial precarity. Gilsanz and colleagues (2019) examined midlife factors that are associated with dementia risk and found that experiencing at least one midlife stressor, such as marital stress, financial strain, or employment difficulties, was associated with an elevated dementia risk. If older adult GED recipients experienced greater midlife stressors than high school graduates, this may partly explain their increased odds of cognitive impairment (Gilsanz et al., 2019).

### Hearing and Vision Impairment

The current study found that older adult GED recipients have higher odds of hearing impairment and vision impairment than older adults with high school diplomas. An important factor that may increase hearing loss in older age is noise exposure in occupational settings (Cruickshanks et al., 2003). Midlife noise exposure may cause subclinical damage, in which the damage is not immediately apparent at the time to the individual but still predisposes them to develop hearing loss in later life (Cruickshanks et al., 2010). Occupations with high levels of noise exposure are generally associated with lower levels of education (Cruickshanks et al., 2010). The differences in hearing impairment between GED recipients and high school graduates may partly result from differential occupational outcomes for these two groups. Another possible explanation for the higher odds of hearing impairment among GED recipients is the high number of veterans who received their GED. Although the GED has expanded in the decades since its inception, its origins were to support veterans returning from war. Given the older age of the current sample, there may be a high number of veteran GED recipients in the present study. Veterans have been found to be 30% more likely than nonveterans to have severe hearing impairment (Centers for Disease Control and Prevention [CDC], 2011).

The finding that GED recipients had higher odds of vision impairment supports previous research by Zajacova (2012) that identified a higher prevalence of vision problems among GED recipients when compared to high school graduates. Vision impairment can occur in older adulthood as a complication of other chronic health conditions, such as diabetes (Pelletier et al., 2016). GED recipients have been found to have a higher prevalence of diabetes when compared to high school graduates (Zajacova, 2012). GED recipients have also been found to be less likely to have health insurance than their high school graduate peers (Song & Hsu, 2008). Americans without health insurance are less likely to use eye care services (Zhang et al., 2008). This may result in GED recipients having worse vision outcomes by the time they become older adults because they face greater barriers to accessing eye care services across their lifespan which could help address the progression of their vision impairment.

### ADL Limitations and Ambulation Limitations

The current study found that GED recipients had higher odds of experiencing functional impairments, such as ADL limitations and ambulation limitations, when compared to high school graduates. This aligns with other studies that have found GED recipients to be more likely to experience functional limitations than high school graduates (Liu et al., 2013; Zajacova, 2012). Education is a well-established social determinant of functional decline in later life (Martin et al., 2010). The current study emphasizes that the assumed equivalency between high school graduates and GED recipients neglects the fact that GED recipients often have worse outcomes in areas such as employment and wealth, which are strongly associated with better physical functioning in later life (Zajacova, 2012). Additionally, lower socioeconomic status jobs are often more physically demanding than higher status jobs. If older adult GED recipients had to work more laborious occupations than their high school graduate counterparts in their young adulthood and in midlife, they may be more vulnerable to worse physical functioning in later life. Furthermore, there are other factors that are associated with greater functional limitations in older age, such as having multiple chronic health conditions and smoking (Hajek & König, 2016), both of which are more prevalent among GED recipients than high school graduates (Kurti et al., 2016; Zajacova, 2012).

### Limitations

The findings of the current study should be considered in light of some limitations. First, this study uses self-report data to measure each health outcome. Self-report data may be less accurate than other formal measurements, such as using audiometric measures for hearing impairment or standardized tools for cognitive impairment. However, self-report data are generally easier to gather and far less expensive, facilitating data collection from this study's large sample size. Second, the current study utilized single-question indicators to measure cognitive impairment, hearing impairment, vision impairment, limitations in ADLs, and ambulation limitations, which may be less preferable than more comprehensive disability indicators, such as performance-based tests or medical examinations. However, the measures used in the current study are frequently utilized and have been determined to be reliable and valid disability indicators for large population-based data (Altman et al., 2017; Brault et al., 2007). Third, information on important factors associated with later-life health, such as early life adversities, smoking history, substance use, obesity, physical activity, access to healthcare, and other medical history, was not available in the ACS. Fourth, because we only had data at one point in time, we cannot untangle the age, cohort, and period effects in the current study. It is possible that the observed patterns may or may not be due to the described cohort influences. Fifth, due to the cross-sectional design of the current study, there is no way to assume causality between educational attainment and the examined health outcomes. Lastly, the ACS does not include data on why respondents obtained a GED. This may have implications for health outcomes in later life, such as respondents who left school due to situations of maltreatment and neglect at home, since early adversities are strongly associated with worse health across the lifespan (Felitti et al., 2019; Ritchie et al., 2011).

## Conclusion

Despite these limitations, the current study makes a substantial contribution to the limited body of research examining the health outcomes of older GED recipients and documenting the significant disparities that exist between GED recipients and high school graduates. While GED recipients had better health outcomes than high school dropouts in cognitive impairment, hearing impairment, vision impairment, ADL limitations, and ambulation limitations, their health status was not equivalent to that of their high school graduate peers for any of the examined health outcomes. This study indicates a gradient in health outcomes by educational attainment among American older adults and underlines the importance, where possible, of separating GED recipients from high school graduates in future research.
